# Impact of poverty on hypertension and cardiovascular disease in sub-Saharan Africa

**Published:** 2007-07

**Authors:** YK Seedat

**Affiliations:** Nelson R Mandela School of Medicine, Faculty of Health Sciences, University of KwaZulu-Natal, Durban

## Abstract

**Summary:**

Sub-Saharan Africa (SSA) has huge amounts of natural resources and a source of strategic minerals. It is not overpopulated compared to the Asian continent, yet the economic conditions have deteriorated alarmingly in recent years. It is the poorest continent and has the lowest per capita income in the world. An analysis of the causes of poverty and its impact on health, particularly cardiovascular diseases (CVD) and hypertension, was carried out and is reported on here.

A ‘second-wave epidemic’ is currently sweeping through SSA, other developing countries and Eastern Europe, making a comprehensive CVD programme necessary. Social, economic and cultural factors impair the control of hypertension, diabetes, obesity, tobacco use and other risk factors for CVD in SSA. Primary prevention through a population-based, lifestyle-linked programme, as well as cost-effective methods for detection and management are synergistically linked. The existing healthcare infrastructure needs to be orientated to meet the challenge of CVD, while empowering the community through health education.

## Summary

In 1929 an article appeared in the *Lancet* describing blood pressure in an extremely primitive rural African community. Dennison wrote, ‘Over two years ago at a native hospital in the south of Kavirondo in Kenya, during which period approximately 1 800 patients were admitted, no case of raised blood pressure was encountered, although low blood pressure was not uncommonly encountered. On no occasion was a nephrosclerosis or interstitial nephritis made’.^1^

## Why is Africa poor?

This must be examined from a historical and social aspect. Africa was involved in slavery between 1441 and 1870. This was followed by colonisation by Belgium, Britain, Germany, Holland, France, Italy, Portugal and Spain. Between the 1960s and 1991 we entered the ‘cold war’ and colonial powers retreated from Africa. Foreign powers adopted self-interest policies and sold arms to despot rulers in Africa. African leaders indulged in fraud/embezzlement, which was tolerated by the Western powers who marginalised Africa from the benefits of globalisation. Corruption has spread among the populations of Africa.^2^

Today one finds that sub-Saharan Africa is the poorest continent in the world. Agricultural output has decreased due to drought, outdated agricultural equipment, political interference and diminished agricultural export to Europe because of subsidisation. 3 The healthcare expenditure in many of the 54 countries in SSA is around US$10 per person annually in contrast to between US$2 000 and $5 000 in industrialised Western countries. Africa has a huge debt and the interest on the debt is equal to twice the amount spent on health and education. The Third World debt is a modern version of the exploitation characteristic of the slave-trade era.^2^

According to Wikipedia, the free encyclopedia (http://www.google.com), the Gini coefficient is a measure of statistical dispersion, most prominently used as a gauge of inequality of income or wealth distribution. It is defined as a ratio with values between 0 and 1: the numerator is the area between the Lorenz curve of the distribution and the uniform distribution line; the denominator is the area under the uniform distribution line. Therefore, a low Gini coefficient indicates more equal income or wealth distribution, while a high Gini coefficient indicates unequal distribution; 0 corresponds to perfect equality (everyone has the same income) and 1 corresponds to perfect inequality (one person has all the income, while everyone else has zero income). The Gini coefficient requires that no one has a negative net income or wealth.

The Gini coefficient was developed by the Italian statistician Corrado Gini and published in his 1912 article ‘Variabilita e mutabilitas’ (Variability and mutability). The Gini index is the Gini coefficient expressed as a percentage, and is equal to the Gini coefficient multiplied by 100. The Gini per capita index in SSA in 2000 was US$470 and in most countries of the developing world it was below US$5 000, whereas it was US$40 000 in the US and Switzerland. The Gini index showed that the lower the income of a country the lower the educational status and life expectancy.^4^

## Health status in Africa and globally

In 1994 the World Bank estimated that one billion people globally live in absolute poverty, 900 million are illiterate, two billion are without potable water, 100 million are homeless, 800 million go hungry every day and 150 million under five years old are undernourished. Twenty per cent of the world’s population controls 80% of the resources and wealth.^5^

The number of poor people in Africa, defined as those making less than a dollar a day, has increased sharply in both relative and absolute terms. The absolute number of poor people has grown five times more than the figure for Latin America and twice that for South Asia. For example, in 1995 the population of Africa was estimated to be 580 million. Of these, 291 million had average incomes of below one dollar a day in 1998; 124 million of those up to the age of 39 years were at risk of dying before age 40; 43 million were stunted as a result of malnutrition; 205 million were estimated to be without access to health services in the 1990−1995 period; 249 million were without drinking water in the 1990−1995 period; and more than two million died annually before their first birthday.^6^

An assessment of the world’s priorities and the cost in billions of US$ is shown in Table 1.^7^ This compares the amount which the First World spends and ‘squanders’, with the amount estimated to be needed to provide basic requirements for the developing world.

**Table 1:** The World’s Priorities: Annual Expenditure

*Basic education for all*	$6 billion
Cosmetics in the United States	$8 billion
*Safe water and sanitation for all*	$9 billion
Ice cream in Europe	$11 billion
*Reproductive health for all women*	$12 billion
Perfumes in Europe and the United States	$12 billion
*Basic health and nutrition*	$13 billion
Pet food in Europe and the United States	$17 billion
Business entertainment in Japan	$35 billion
Cigarettes in Europe	$50 billion
Alcoholic drinks in Europe	$105 billion
Narcotic drugs in the world	$400 billion
Military spending in the world	$780 billion

Items highlighted denote the necessity globally. Source: Human Development Report, 1998. 7

The WHO Global Status of Health found that the Americas have 10% of the global burden of diseases and utilise 38% of the world’s healthcare workers for 50% of the world’s diseases. In Africa, 11% of the world’s population has 24% of the world’s diseases but only 3% of the world’s healthcare workers and just 1% of the global financial resources. The report identified 57 countries that cannot meet a widely accepted basic standard for healthcare; 36 of these ‘critical countries’ are in SSA.

Critical shortage was defined as insufficient numbers of doctors, nurses and midwives to achieve an 80% coverage rate for deliveries by skilled birth attendants or for measles immunisation. WHO estimates that it would cost an average of US$136 million per country annually to meet these needs, necessitating an average increase in healthcare expenditure of US$2.80 per person per year. But these figures focus on only doctors, nurses and midwives. They don’t take into account emigration of workers, or payment of salaries, which would cost an additional US$311 million per country. The staff shortage in SSA derives from a combination of underproduction of qualified healthcare workers, internal maldistribution of professionals and emigration of trained workers.^8^

## Population of world and sub-Saharan Africa

The world’s population is rapidly escalating. It is estimated that at Rome’s peak, the global population was 200 million, and at around AD 1500 it was 400 million. During the industrial revolution it was one billion and the current world population is six billion. It is estimated that by 2050 the population will be nine billion. SSA has a population of about 750 million and it is increasing, in spite of a high death rate from HIV/AIDS.

There is rapid urbanisation globally and in 1994, the rate was 44.5%; in 2025 it is projected to be 61.1%. Developing countries showed a rise in urbanisation from 12.6% in 1970 to 21.9% in 1994 and it is projected to be 43.5% in 2025. Economies in transition had an urbanisation rate of 25.1% in 1970, rising to 37% in 1994 and projected to be 57% in 2025. The rapid urbanisation in SSA and formation of peri-urban segments has resulted in the development of slums.

## Cardiovascular disease

This accounts for 16.7 million or 29.2% of the total global deaths. Around 80% of cardiovascular deaths take place in low-and middle-income countries. At least 20 million people survive heart attacks and strokes each year, many requiring costly clinical care.9 In developing countries, cardiovascular disease (CVD) predominantly affects people of working age (30–64 years). The disability in middle age has major social and economic consequences. As healthcare improves for the wealthy, there is a reversal in the socio-economic gradient, and poorer and disadvantaged people suffer the largest burden of CVD. This has already occurred in the developed world and seems to be manifesting in middle- and low-income countries, especially for risk factors for CVD, which are predictors for future events.

Prevention and treatment of risk factors for CVD are effective and sustainable in the long run. The most important risk factors for CVD are hypertension, high cholesterol levels, tobacco usage and diabetes. Sixty per cent of the burden of cardiovascular disease and about half of coronary heart disease globally is caused by hypertension. The major modifiable risk factors for CVD are high salt intake, increased body weight and physical inactivity.^9^ Hyperlipidaemia is not as yet a major risk factor in SSA black populations but it is on the increase.^10^

In the Interheart African study, the risk of acute myocardial infarction (AMI) increased with higher income and education levels in the black African group, in contrast to the findings in other racial groups such as whites and coloureds.11 A history of hypertension in patients revealed higher rates of myocardial infarction (MI) in the black African group than in subjects in the Interheart study overall. The authors concluded that known CVD risk factors account for approximately 90% of the MI observed in the African population,^11^ which is consistent with results of the overall Interheart study.^12^ Contrasting gradients of risk-factor patterns and AMI risk found in different socio-economic classes of the various ethnic groups suggest that they are at different stages of the epidemiological transition.^11^

## Impact of poverty on hypertension

Hypertension is more common in the urban than the rural population. Migratory studies have shown that hypertension in Kenya was associated with a rise in blood pressure as the migrants moved from a rural to an urban setting. The urban migrants had higher body weights, pulse rates and urinary sodium:potassium ratios than did those that remained in the rural areas. The higher pulse rates in the Nairobi participants also suggested that increased autonomic nervous system activity could have contributed to the higher blood pressure levels.^13^

Opie and Seedat suggested the following hypothesis to explain the high prevalence of hypertension in the urban black population. The subjects had: low plasma renin values; sodium hypersensitivity and cellular abnormalities; increased epithelial sodium channels; different genes controlling the renin angiotensin aldosterone system; increased peripheral vascular resistance; obesity; low socio-economic status and the underweight phenotype.^14^

Obesity is associated with increased blood pressure levels in blacks.^15^ In the South African Demographic and Health survey of 1998, the prevalence of obesity (body mass index ≥ 30 kg/m^2^) was 30% in black females and 8% in black males.^15^ It has been suggested that in Africa the rural diet is relatively healthy, but with urbanisation, the diet is replaced with a higher fat and lower carbohydrate intake.^16^ Poverty and cultural factors hinder the implementation of the DASH (Dietary Approaches to Stop Hypertension) high-fruit, high-vegetable, low-salt diet, which was found to be very effective in African−Americans.^17^ In Tanzania, in 9 254 urban inhabitants of Dar-es-Salaam, socio-economic status was inversely related to blood pressure levels and smoking, and increasing affluence to increased obesity.^18^ African urbanisation is associated with more stress, dietary changes and acculturation.^10^,^13^

## Clinical features

The commonest complication of hypertension is stroke, and hypertension and diabetes rates are increasing in Africa.^10^,^14^,^19^ Even rural communities are not spared the scourge of stroke. In a recent study of a South African semi-rural community, the prevalence of stroke was relatively high, with a rate of stroke disability approaching that in the high-income countries.^20^ In a review of stroke epidemiology from population-based studies of incidence, prevalence and case fatality, it was found that there were limited studies on stroke occurrence in Africa and they did not meet the criteria for an ‘ideal’ stroke study.21 It is estimated that there were roughly 79.8 million people with stroke in SSA in 2000 and this incidence is projected to rise to 150.9 million by 2025.^22^

Congestive heart failure is the commonest cardiac manifestation of hypertension,^23^ although coronary heart disease is still relatively uncommon, probably because of low serum cholesterol and high HDL cholesterol levels in the SSA populations. 24 However, atheroma of the aorta and cerebral arteries is common,^25^ and renal failure from malignant hypertension is not uncommon.^26^

## Effects of poverty on the prevention and treatment of hypertension

The estimated prevalence of hypertension in SSA in 2000 was 41.6 million males and 38.2 million females. It is estimated the prevalence of hypertension globally is 950 million people out of a world population of 6.2 billion. About 65% of hypertensive patients are in the developing world. Racial group implies a common gene. Ethnic group implies sharing a cultural identity, ie, biological similarity plus shared social and lifestyle values. The major ethnic groups in SSA are white Caucasian: two billion people, South Asian: 1.2 billion, Chinese: 1.5 billion, black African: 0.7 billion, Arab: 0.3 billion, and others: 0.5 billion.

In spite of effective drug treatment available for hypertensive patients in general, economics with regard to the cost:benefit ratio and social considerations continue to influence the low rate of detection, treatment and control of hypertension in the black population of Africa and the USA.

Over the next few years, there will be a rise in the number of cardiovascular deaths in developing countries, including SSA. This is expected to be due to:

● a decline in deaths in infancy, childhood and adolescence from infections
● industrialisation and urbanisation
● change from high-fibre to a low-fibre diets
● low birth weights in babies, producing metabolic abnormalities (‘foetal origins hypothesis’ of Barker). The Barker hypothesis suggests that disturbed intrauterine growth has a negative influence on the development of the cardiovascular system and favours the occurrence of hypertension, diabetes mellitus, insulin resistance, hypercholesterolaemia, and hyperuricaemia in adult life. Many chronic disorders that manifest later in life and may be related to two seemingly opposing factors potentially present early in life: poverty [ie, malnourished mothers give birth to malnourished infants with low birth weights (LBW)]; and prosperity [exposure of an infant with LBW phenotype to a high-energy (caloric) diet]. These factors contribute to the biological phenomenon of developmental plasticity, or the ability of a genotype to produce multiple forms and behaviours in response to environmental conditioning
● increase in tobacco use.

From 1990 to 2020, there is a change predicted in the rank orders of disease burden for 15 leading causes of disability and death in the world, as shown by DALYS (disability in life years). Ischaemic heart disease will be the commonest cause of DALYS in 2020.^27^ Accurate figures of deaths in SSA are not available.

The South African Health Review has found a steady decline in the health of the South African population in recent years. HIV/AIDS accounted for 39% of all deaths in 2000 and diseases of lifestyle (heart disease, stroke, cancer, chronic obstructive pulmonary disease, hypertension and diabetes) accounted for 38% of all deaths. Other causes of deaths were homicide and violence (7.5%), tuberculosis (5%) and road accidents (4.1%).^28^

Developing countries, particularly SSA, are financially unable to intervene in all the risk factors for CVD. Extensive investigations and costly drugs are not readily available and are probably inadvisable. SSA has the burden of pre-transitional diseases such as infections and nutritional disorders, and post-transitional diseases such as CVD.^10^

Strategies for the prevention of CVD in SSA are:

● In primordial prevention, the main objective is to prevent the acquisition or enhancement of CVD risk factors. This is done by avoidance or decrease of the social, economic and cultural determinants that contribute to the development of hypertension. Primordial prevention relies on health policies that create a congenial environment to promote healthy behaviour and population-wide education programmes. In turn, they depend on many factors including political commitment and the involvement of political leaders and the mass media.
● Primary prevention aims to reduce or reverse the risk factors in urban communities. This is done by reducing or modifying the risk factors of hypertension through appropriate policies and educational programmes, in order to prevent or delay the development of hypertension. The resultant change in behaviour must take place at the population level, for example, low salt intake or increased physical activity can produce benefits across the whole spectrum of blood pressure distribution. Since a substantial number of adults have blood pressure above the optimal level, even a small reduction in blood pressure can bring about a significant decrease in cardiovascular risk in the population. At the individual level, primary prevention of hypertension consists of adopting healthy lifestyles at an early age.
● There should be non-pharmacological, population-based and lifestyle-linked measures, such as salt restriction, exercise, control of obesty, increased potassium intake in the form of fresh fruit and vegetables and stopping cigarette smoking.
● One should develop cost-effective methods for the diagnosis of hypertension and low-cost saving measures, such as microscopic urine examination and testing for urinary albumin and glucose, and serum creatinine, potassium and blood sugar levels.
● At a tertiary level one should avoid high-cost, low-yield technologies, such as routine echocardiography for hypertension, and computerised resonance tomography and magnetic resonance tomography for renovascular disease.^10^

The reasons that national policies for prevention of CVD have not yet emerged in developing countries are:

● Competing priorities: infectious and nutritional disorders are still major health challenges and HIV/AIDS takes up a large portion of healthcare resources.
● Technology-based resource interventions are favoured by policy makers and the media. The proximity of respected clinical cardiologists to policy makers who belong to the social class most affected by CVD often results in iniquitous resource allocation.
● There is inadequate reliable epidemiological data on the burden of the disease and the distribution of risk factors.
● Healthcare workers may not present the situation to policy makers and the media adequately.
● Discordant messages: false claims are made that risk factors for CVD are not important.
● Failure to recognise the importance of prevention and cost effectiveness: many health professionals are unconcerned about preventive action because of lack of epidemiological orientation during training, the pressures of providing healthcare in crowded clinics, and the lack of concern about cost effectiveness.
● Anonymity: health professionals receive no direct recognition from their beneficiaries for their endeavours, as they do when providing healthcare.
● Economic and social constraints: dietary advice, although scientifically proven, may be irrelevant or impractical for reasons of availability, affordability and acceptability of foodstuffs.
● Vested interests: tobacco and salt companies influence policy makers and the media.
● There is a lack of community mobilisation.
● No concerted efforts have been made to educate the community about the risk factors and dangers of CVD.
● There is a failure to utilise non-medical personnel in the detection and treatment of CVD and in facilitating adherence to prescribed medication for CVD.
● There is failure to initiate measures for the coordination of actions to prevent CVD among various stakeholders and the private sector.^10^

There is evidence that the prevalence of hypertension and CVD is increasing rapidly in SSA. Two recent surveys indicated that in Tanzania just under 20% of hypertensive subjects were aware of their diagnosis, approximately 10% reported receiving treatment and less than 1% were controlled (BP < 140/90 mmHg).^29^ The treatment status of South African black males showed that 20% were aware of their hypertension, 14% were on treatment and only 7% were controlled (BP < 140/90 mmHg). For females, 47% were aware of their hypertension, 29% were on treatment and only 15% were controlled.^30^

While it is important to consider the science of medicine for the treatment of hypertension, particular consideration should be given to cost-effectiveness and affordability, as many countries in SSA have severe resource constraints. In some countries, the health budget per capita does not exceed US$10 per year and is completely insufficient to address the needs posed by the double burden of non-communicable and infectious diseases, including AIDS. Guidelines for the prevention, treatment and control of hypertension for SSA have been published by the International Forum for Hypertension Control and Prevention in Africa.^31^

## Conclusion

The treatment of hypertension in a poverty stricken continent such as SSA presents a challenge. The current approaches to prevent most premature CVD are at the individual level and focus on high-risk factors, but these are too little, too few, and too late. The future approach to CVD prevention should be aimed at the population level on societal change, that is, throughout the life of the individual, and on large changes in multiple risk factors. We should remember the wise words of Nelson Mandela – ‘We must face the matter squarely, that where there is something wrong in how we govern ourselves, it must be said that the fault is not in the stars, but in ourselves. We know that we have it in ourselves as Africans to change all this. We must assert our will to do so − we must say that there is no obstacle enough to stop us from bringing about an African renaissance.’
